# The regulation of autophagy differentially affects *Trypanosoma cruzi* metacyclogenesis

**DOI:** 10.1371/journal.pntd.0006049

**Published:** 2017-11-01

**Authors:** María Cristina Vanrell, Antonella Denisse Losinno, Juan Agustín Cueto, Darío Balcazar, Laura Virginia Fraccaroli, Carolina Carrillo, Patricia Silvia Romano

**Affiliations:** 1 Laboratorio de Biología de *Trypanosoma cruzi* y la célula hospedadora. Instituto de Histología y Embriología (IHEM), Facultad de Ciencias Médicas, Universidad Nacional de Cuyo-CONICET, Mendoza, Argentina; 2 Instituto de Ciencias y Tecnología Dr. César Milstein—CONICET; Buenos Aires, Argentina; Instituto de Investigaciones Biotecnológicas, ARGENTINA

## Abstract

Autophagy is a cellular process required for the removal of aged organelles and cytosolic components through lysosomal degradation. All types of eukaryotic cells from yeasts to mammalian cells have the machinery to activate autophagy as a result of many physiological and pathological situations. The most frequent stimulus of autophagy is starvation and the result, in this case, is the fast generation of utilizable food (e.g. amino acids and basic nutrients) to maintain the vital biological processes. In some organisms, starvation also triggers other associated processes such as differentiation. The protozoan parasite *Trypanosoma cruzi* undergoes a series of differentiation processes throughout its complex life cycle. Although not all autophagic genes have been identified in the *T*. *cruzi* genome, previous works have demonstrated the presence of essential autophagic-related proteins. Under starvation conditions, *Tc*Atg8, which is the parasite homolog of Atg8/LC3 in other organisms, is located in autophagosome-like vesicles. In this work, we have characterized the autophagic pathway during *T*. *cruzi* differentiation from the epimastigote to metacyclic trypomastigote form, a process called metacyclogenesis. We demonstrated that autophagy is stimulated during metacyclogenesis and that the induction of autophagy promotes this process. Moreover, with exception of bafilomycin, other classical autophagy modulators have similar effects on *T*. *cruzi* autophagy. We also showed that spermidine and related polyamines can positively regulate parasite autophagy and differentiation. We concluded that both polyamine metabolism and autophagy are key processes during *T*. *cruzi* metacyclogenesis that could be exploited as drug targets to avoid the parasite cycle progression.

## Author summary

In spite of its old discovery, more than one hundred years ago, *Trypanosoma cruzi*, the causative agent of Chagas’ disease, is still prevalent in the world, infecting more than 6 million people mostly in Latin America, where this illness is endemic. Only two approved drugs, benznidazole and nifurtimox, are currently used for the treatment of Chagas’ disease. Although efficient for the acute phase, they are poorly effective in the chronic period of the disease and they cause many undesirable side effects. There is an urgent need for therapeutic alternatives. To this end, identifying and validating novel molecular targets is critically relevant. This study describes the effect of different inhibitors on the *T*. *cruzi* autophagic pathway, a process required for parasite differentiation. Herein, we demonstrate that the regulation of parasite autophagy exhibits similarities and differences with host cell autophagy. Our study provides new insights that could be used to avoid *T*. *cruzi* cycle progression in both insect and mammalian hosts.

## Introduction

Autophagy is a major intracellular degradation/recycling system ubiquitous in eukaryotic cells. It contributes to the turnover of cellular components by delivering portions of the cytoplasm and organelles to lysosomes, where they are digested [1]. Depending on the mechanisms used for the delivery of cargo to lysosomes, three different types of autophagy have been described in mammalian cells: macroautophagy, microautophagy, and chaperone-mediated autophagy (CMA) [2]. Macroautophagy, referred to as autophagy in the rest of this work, involves a first step of autophagosome formation followed by autophagosome maturation. Initially, the cytoplasmic materials are sequestered by the phagophore, a curved membrane that elongates around the cargo to form a double membrane vesicle called autophagosome. Autophagosomes next interact with endocytic compartments and finally fuse with lysosomes to form autolysosomes where the enclosed materials are hydrolyzed [1]. Several genes required for autophagy have been described. Their products, the so-called Autophagy (Atg)-related proteins, comprise the core molecular machinery responsible for the sequential activation of this pathway [3]. The Atg8 protein (or LC3 in mammalian cells), is the best marker of autophagy. Atg8 is present in the membrane of all compartments of this pathway, from the phagophore to the autolysosome [4]. The formation of autophagosomes and execution of autophagy critically depend on proteolytic processing of Atg8 by the cysteine protease Atg4, and its subsequent conjugation to the phosphatidylethanolamine in the expanding phagophore membrane [5].

It is known that two major kinases differentially regulate mammalian autophagy: the mammalian target of rapamycin (mTOR) and the class III PI3K Vps34. mTOR is an evolutionary conserved kinase that senses the nutrient and energy status of cells by forming two distinct complexes. One of them, mTORC1 enhances glycolysis and biosynthetic processes and inhibits autophagy [6]. Therefore, inhibition of mTORC1 by treatment with rapamycin (Rap), an immunosuppressive drug, results in a potent induction of autophagy. In contrast, the activity of Vps34 is essential for autophagy. In mammalian cells Vps34 forms a complex with beclin-1 (the mammalian ortholog of yeast Atg6) and other proteins to promote the production of phosphatidylinositol 3-phosphate, thereby facilitating lipid membrane changes required for autophagosome formation and maturation [7]. The PI3K inhibitor wortmannin (Wort) has been widely used to inhibit yeast and mammalian autophagy for its inhibitory action on the beclin-1/Vps34 complex [8].

The polyamine spermidine (Spd) has been recently described as a new modulator of autophagy since Spd inhibits the activity of histone acetyl transferase, leading to the upregulation of several *ATG* genes including *ATG7*, *ATG11* and *ATG15* [9]. When added to culture media, Spd is also able to directly induce autophagy in a transcription-independent manner. The mechanism has not been fully elucidated yet; however, this phenomenon could be due to the enhanced deacetylation of essential autophagy-related proteins such as ATG5 and ATG7 [10]. Furthermore, the same concentrations of Spd that exert proautophagic effects also have a marked life span-extending action on yeast, nematodes and flies. Conversely, the genetic inhibition of essential *ATG* genes abrogates the life span extension induced by Spd, indicating that this polyamine can prolong the life span by the induction of autophagy [9].

The parasitic protozoan *Trypanosoma cruzi*, which is the causative agent of Chagas’ disease, presents four well differentiated stages in its complex life cycle, which alternates between insect vectors and mammalian hosts. The bloodsucking triatomine bugs acquire the parasites by ingestion during a blood meal of an infected mammalian host. A few hours after the meal, in the anterior region of the midgut, bloodstream trypomastigotes transform into proliferative, non-infective epimastigotes. After several rounds of replication, epimastigotes transform into the non-proliferative, infective metacyclic trypomastigotes (MT), a process called metacyclogenesis. MT are released along with the feces and urine of the insect and may infect a new mammalian host. Firstly, MT infect macrophages and epithelial cells in the site of entry and then cardiac and smooth muscle fibers that are the major targets of *T*. *cruzi*. The infection of these cells is responsible for the main clinical manifestations of the disease. Inside the cell, the parasite undergoes another dramatic transformation into proliferative intracellular amastigotes. After intense multiplication in the host cell cytoplasm, amastigotes transform into bloodstream trypomastigotes that can infect other neighboring cells or reach the circulatory system, thus completing the cycle [11].

Autophagy also occurs in trypanosomatid parasites. Half of the known yeast and mammalian ATG proteins have also been found in vertebrate pathogenic trypanosomatids (*Trypanosoma brucei*, *Trypanosoma cruzi* and *Leishmania* spp.), although with low sequence conservation [12]. More than one *ATG8* gene was identified in the Trypanosomatidae family: three in *T*. *brucei*, two in *T*. *cruzi* and, unexpectedly, four families comprising together 25 genes in *Leishmania major*. *T*. *cruzi* has a ‘true’ *Tc*ATG8.1 and a *Tc*ATG8.2, which does not seem to participate in autophagy [13]. *T*. *cruzi* also contains two *ATG4* isoforms, *TcATG4*.*1* and *TcATG4*.*2* whose products, which are called autophagins, are in charge of ATG8.1 processing [14].

The cellular remodeling during differentiation is essential for the progression of the life cycle of many unicellular eukaryotic pathogens such as *Leishmania* spp. [15], and *T*. *cruzi* [16]; however, the mechanisms involved in these processes have not been fully characterized. The first morphological indications that autophagy occurs during differentiation of trypanosomatids were provided by electron microscopy images of *T*. *brucei* taken by Vickerman and colleagues in the 1970s [17]. Long after that, molecular studies corroborated the presence of ATG genes in trypanosomatids and their participation in the differentiation processes [12,13,18]. Although ultrastructural and molecular approaches have demonstrated the existence of autophagosomes in *T*. *cruzi* [13], a functional characterization of this pathway is still lacking.

Metacyclogenesis of *T*. *cruzi* takes place in the insect's rectum due to many factors such as nutrient scarcity produced by the fast replication of epimastigotes, specific components of intestinal wall and lumen of the vector, etc. The *in vitro* stimulation of this process has been achieved in aged cultures of epimastigotes [19] or by thermic and nutrient stress [20,21] as explained below. Regardless of the method, nutrient deprivation, the classical inducer of autophagy, is the common most frequent stimulus required to trigger epimastigote differentiation. In this report, we made a functional analysis of *T*. *cruzi* autophagy under different conditions. We have observed that autophagy is induced during *T*. *cruzi* metacyclogenesis and that different drugs known to regulate mammalian autophagy exert either a positive or a negative effect on parasite autophagy and differentiation. We have also shown that similarly to mammalian cells, spermidine and related polyamines are important inducers of parasite autophagy and that mutant parasites that produce their own polyamines display higher autophagic activity and higher metacyclogenesis efficiency, as compared to wild type parasites. Taken together, these data demonstrate the key role of autophagy for *T*. *cruzi* differentiation and highlight a new target that could be used to interrupt the *T*. *cruzi* cycle progression.

## Methods

### Ethical statement

We are cognizant of the Argentinean (ANMAT 5330/97) and international (Declaration of Helsinki) principles and bioethical codes, and guarantee that all procedures carried out in conducting the research reported here were in compliance with both. Human subjects were involved in this project for the purpose of sera donation. The subject population consisted of healthy male donors, 25 year of age or over, which signed a written Informed Consent form at the time of their enrollment. The Research Committee of the Central Hospital of Mendoza and the Bioethical Committee of the Diego Paroissien Hospital of Mendoza (Comité de Investigación del Hospital Central de Mendoza, President: Dr Carlos Zanessi y Comité de Bioética del Hospital Diego Paroissien de Mendoza; President: Dr Jorge Sotile) approved our protocol for the collection and manipulation of human serum samples. All laboratory procedures followed the safety regulations of the Hospitals and Medical School.

### Media

TAU medium was prepared with 190 mM NaCl (Biopack), 17 mM KCl (Biopack), 2 mM MgCl_2_ (Biopack), 2 mM CaCl_2_ (Biopack) and 8 mM sodium phosphate buffer (pH 6 to 6.8). Modified TAU medium (TAU-AAG) was prepared with TAU medium supplemented with 50 mM sodium glutamate (Sigma), 10 mM L-proline (Tetrahedron), 2 mM sodium aspartate (Sigma), and 10 mM glucose (Biopack). Diamond medium contains 6.25 g/l tryptose (Sigma), 6.25 g/l tryptone (Sigma), 6.25 g/l yeast extract (Sigma), 7.16 g/l KH2PO4 (Biopack) (pH 7.2) and 6.66 mM hemin (Calbiochem) prepared in 3 ml 1N NaOH (Tetrahedron) and 20 ml 1M Tris HCl (Tetrahedron) (pH 6.8). BHT medium was prepared with 33 g/l Brain heart infusion broth (Britania), 3 g/l tryptose, 0.4 g/l KCl, 0.3 g/l glucose and 3.2 g/l Na_2_HPO_4_ (Biopack). SDM79 medium, which contains only traces of polyamines, was prepared with 8.4 g/l 199 TC 45 medium (Sigma), 8 ml/l MEM amino acids 50x (Gibco), s/c L-glutamine (Carbiochem), 6 ml/l MEM Non-essential amino acids 100x (Gibco), 1 g/l glucose, 8 g/l HEPES (Carbiochem), 5 g/l MOPS (Carbiochem), 2 g/l NaHCO_3_ (Biopack), 100 mg/l sodium pyruvate (Sigma), 200 mg/l L-alanine (Tetrahedron), 100 mg/l L-arginine (Sigma), 300 mg/l L-glutamine (Sigma), 70 mg/l L-methionine (Sigma), 80 mg/l L-phenylalanine (Sigma), 600 mg/l L-proline (Sigma), 60 mg/l L-serine (Tetrahedrum), 160 mg/l L-taurine (Sigma), 350 mg/l L-threonine (Sigma), 100 mg/l L-tyrosine (Sigma), 10 mg/l adenosine (Sigma), 10 mg/l guanosine (Sigma), 50 mg/l glucosamine-HCl (Sigma), 4 mg/l folic acid (Sigma), (pH 7,3).

### Parasites

Epimastigotes of Y or Y-GFP strain were cultured in Diamond medium with 10% fetal bovine serum (Natocor) at 28°C. Y-GFP-ODC [22] and Y-GFP-PAT12 [23] mutants co-expressing GFP and the ornithine decarboxylase gene (ODC) (AN Y08233.1) or the PA transporter PAT12 (AN AY526253, also annotated as FJ204167) respectively were maintained in the semisynthetic medium SDM79, to select auxotrophy at 28°C. All cultures contain 20 mg/l hemin (Calbiochem), 10% inactivated fetal bovine serum, 250 μg/ml geneticin (Gibco) for GFP selection, 100 mg/ml streptomycin (Gibco) and 100 U/ml penicillin (Gibco).

### *T*. *cruzi* differentiation protocol

To induce *T*. *cruzi* metacyclogenesis we performed a previously published *in vitro* protocol schematized in S1 Fig [20,21]. Briefly, epimastigotes of *T*. *cruzi* Y or Y-GFP strain (or the mutants Y-GFP-ODC or Y-PAT12) grown to stationary phase (5 x 10^7^ cells/ml) were collected by centrifugation at 2000 g for 15 min, and resuspended at 5 x 10^8^ cells/ml in TAU medium. After 2 h at 37°C (1st stage of metacyclogenesis), parasite samples were processed for microscopy or molecular studies. Similar procedures were conducted in control parasites maintained in Diamond, BHT or SDM79 medium at 28°C. In other cases, to complete the differentiation process, parasites were diluted 100 times in TAU-AAG or control media and maintained at 28°C for 48 h (2nd stage of metacyclogenesis). After this period, differentiated parasites (MT) were then directly quantified using human fresh serum or used for infection assays (see below).

In some experiments, TAU medium was supplemented with 100 nM wortmannin (Wort, Sigma-Aldrich), 100 nM bafilomycin (Baf, Sigma-Aldrich) or 1 mM difluoromethylornithine (DFMO, Sigma) as autophagy inhibitors. For autophagy induction, 50 ng/μl rapamycin (Rap, LC Laboratories), 100 μM spermidine (Spd, Sigma) or 100 μM spermine (Spm, Sigma) was added to control media.

### Trypan blue dye exclusion test

After the first period of metacyclogenesis parasites were stained with the Trypan blue vital dye to study parasite viability. Control and TAU samples were deposited on coverslips and stained parasites were counted by conventional microscopy. An aliquot of parasites were exposed to UV and used as a positive control of mortality.

### Atg8.1 detection

To study autophagic activity parasites were subjected to the first period of metacyclogenesis and processed to detect autophagosomes by indirect immunofluorescence with a specific antibody against the *Tc*Atg8.1 protein (AN ABH07412) generously given by Dr. Vanina Alvarez (IIB-INTECH UNSAM-CONICET). Briefly, parasites were fixed with 4% paraformaldehyde (Sigma-Aldrich) solution in PBS for 15 min at room temperature, washed with PBS, and quenched with 50 mM NH4Cl (Merck) for 15 min at room temperature. Subsequently, cells were permeabilized with 1% saponin (Sigma-Aldrich) in PBS containing 1% bovine serum albumin (BSA-Sigma), and then incubated with the primary antibody against Atg8.1 (1:500) followed by incubation with Cy-3 (excitation wave: 550 nm and emission wave: 570 nm, ThermoFisher) or Alexa 488 (excitation wave: 490 nm and emission wave: 525 nm, ThermoFisher) conjugated anti-rabbit (1:500) secondary antibodies. After that parasites were mounted on coverslips with mowiol 4–88 reagent (Calbiochem) and examined by confocal microscopy. For colocalization studies, after detection of Atg8.1, parasites were incubated with 10 μg/ml of dequenched BSA (red DQ-BSA, excitation wave: 590 nm and emission wave: 620 nm, Invitrogen) for 2 h, washed three times with PBS and then mounted on coverslips with mowiol before examination.

### Transmission electron microscopy

Epimastigotes were exposed to the first period of metacyclogenesis in Diamond (control) or TAU (starved) medium, during 2 h at 37°C and then fixed and processed by Electron Microscopy. Briefly, parasites were fixed with 2% glutaraldehyde (Ted Pella) in PBS for 2 h at 4°C, washed three times with PBS pH 7.2 and subsequently treated with 1% osmium tetroxide (Ted Pella) for 2 h at 4°C. In a next step, parasites were washed again with PBS and sequentially dehydrated in solutions with increasing concentrations of acetone. Finally, samples were included in the epoxy resin (Spurr) and ultrathin sections in an ultramicrotome Leica Ultracut R were performed. Sections were contrasted with uranyl acetate / acetone for 3 min, washed with distilled water and colored with lead citrate for 2 min before observation with the Zeiss 900 electron microscope.

### Semi-quantitative RT-PCR

*T*. *cruzi* epimastigotes were subjected to the first period of metacyclogenesis in Diamond (control) or TAU (starved) medium during 2 h at 37°C and processed for molecular studies. Parasites were collected and washed and RNA was obtained by TRIzol reagent (ThermoFisher). RT step was performed with Oligo(dT)15 Primer and M-MLV Reverse Transcriptase according to the manufacturer instructions (Promega). Levels of expression of *Tc*Atg8.1 were determined by PCR assay in not saturating conditions using cDNA from both populations of metacyclogenesis induced epimastigotes. Primers 5’-CTTTGGAGCACCGCATCG-3’ (forward) and 5’-CAAAAGTTGCCTCACCCGAG-3’ (reverse) were used to amplify a fragment from the *Tc*Atg8.1 transcript (318 pb); and primers 5’- ATATTTAAACCCATCCAAAATCGAGTAAC-3’ (forward) and 5’- GTCAATTTCTTTAAGTTTCACTCTTGC-3’ (reverse) for 18S rRNA transcript (1029 pb), used as the housekeeping gene. PCR products were separated in 1% agarose gel, stained with SYBR Safe (Invitrogen) and quantified using ImageJ software (http://imagej.nih.gov/). The results were expressed as arbitrary units (AU), normalized to rRNA levels. Data shown represents the mean from 3 independent experiments. Statistical analysis was performed using Student t-test.

### Monodansylcadaverine labeling

Epimastigotes from Y-GFP strain were subjected to the first period of metacyclogenesis in TAU (starvation) or BHT (Control) medium for 2 h at 37°C. Thirty min before the end of incubation, 0.15 mg/ml monodansylcadaverine (MDC, excitation wave: 365 nm and emission wave: 525 nm, Sigma-Aldrich) was added to samples. After that, parasites were centrifuged and washed three times with PBS. Subsequently they were deposited on coverslips previously coated with poly-L-lysine (Merck) and then observed in a confocal microscope Olympus FV 1000 in a thermostatized chamber. Another aliquot of parasites were processed to measure fluorescence intensity in a Multiplate reader. Data were represented using the mean values of percentage of MDC fluorescent parasites and standard errors (SE) of at least three independent experiments. Statistical calculations (Tukey test, * p <0.05, ** p <0.01, *** p <0.001) and graphics were prepared using the software KyPlot.

### DQ-BSA labeling

The method was similar to that of the previous section, with the addition of 10 μg/ml dequenched BSA instead of MDC. This compound emitted red fluorescence after BSA hydrolysis into small peptides in lysosomes, thus identifying degradative compartments. Data were represented using the mean values of percentage of DQ-BSA positive parasites and the error bars indicate SE of at least three independent experiments. Statistical calculations (Tukey test, * p <0.05, ** p <0.01, *** p <0.001) and graphics were performed using the software KyPlot.

### Western blot assay

Epimastigotes from Y strain (15 x 10^6^ cells) were subjected to the first period of metacyclogenesis in BHT medium (control) in the absence or presence of 100 μM spermidine for 2 h at 28°C or in TAU medium (starvation) for 2 h at 37°C. Cells were collected by centrifugation at 2000 g for 15 min, resuspended in sample buffer and incubated for 10 min at 95°C. Protein extracts were run on 18% SDS-PAGE and transferred to Hybond-ECL (Amersham) nitrocellulose membranes. The membranes were blocked in Blotto for 1 h at 4°C (10% non-fat milk, 0.05% Tween 80 in PBS), washed twice with 0.05% Tween 80 in PBS and incubated with a primary antibody anti-LC3 (1:800 dilution, Sigma-Aldrich) followed by a peroxidase-conjugated anti-rabbit secondary antibody (1:10,000 dilution). Anti-Tubulin (1:300 dilution, Developmental Studies Hybridoma Bank) was used to detect Tubulin (AN ESS55047) as a loading control. Detection was accomplished with a chemiluminescence system from Millipore (WBKLS, Biopore, Buenos Aires, Argentina) on a Luminescent Image Analyzer LAS-4000 (Fujifilm, Tokyo, Japan).

### Direct quantification of metacyclic trypomastigotes

To quantify the efficiency of metacyclogenesis at different conditions, parasites were subjected to the first (F) or the complete period (T) of metacyclogenesis in control or TAU medium in the presence of wortmannin (in TAU medium) or rapamycin (in control medium). Subsequently, samples of mixed parasitic forms (epimastigotes / metacyclic trypomastigotes) were centrifuged to remove inhibitors or inducers of the autophagic pathway. Pellets were then resuspended in fresh human serum recently obtained from a healthy male donor, which produces the complement dependent lysis of epimastigotes and facilitates the direct quantification of the serum-resistant metacyclic trypomastigotes in a Neubauer chamber.

### Infection assays

Epimastigotes/ trypomastigotes mixed samples generated as in the previous section were placed on Vero cell (ABAC-Asociación Banco Argentino de Células) monolayers for 24 h at 37°C. After three washes with PBS, to remove non-internalized parasites, cells were fixed with 3% paraformaldehyde for 15 min at room temperature, and quenched with 50 mM NH4Cl in PBS. To facilitate visualization, cellular actin (AN XP_008017958) were stained with rhodamine-conjugated phalloidin (excitation wave: 540 nm and emission wave: 570 nm, Invitrogen) for 1h at 37°C in a humid chamber. Parasite nuclei were visualized in green due to the stable expression of *Tc*H2b histone fused to GFP [24]. Cells were also treated with Hoechst for DNA staining, mounted onto glass slides with Mowiol and analyzed with an Olympus Confocal Microscope FV1000-EVA (Olympus), with the FV10-ASW (version 01.07.00.16) software.

## Results

### Autophagy is induced during *T*. *cruzi* metacyclogenesis

As mentioned in the Introduction, previous works have demonstrated the presence of *ATG* genes in *T*. *cruzi* as well as increased levels of *Tc*Atg8 in parasites undergoing spontaneous differentiation [13]. To better characterize the participation of autophagy during this process, we followed a standardized protocol of differentiation initially published by Contreras et al. [20] and then modified by Ferrari et al [25]. Briefly, epimastigotes were subjected to a short period of nutritional and thermic stress in triatomine artificial urine (TAU) medium at 37°C during 2 h, followed by a chase in TAU medium supplemented with three amino acids (indicated in the Methods section) and glucose (TAU-AAG) during 48 h at 28°C (S1A Fig). The efficiency of this protocol was analyzed by both direct quantification of differentiated MT and by infection assays (see details in the Methods section). Data showed that a significant number of infective forms were obtained from the parasites subjected to TAU compared to parasites maintained in control conditions (see below). After the first period of metacyclogenesis parasites were stained with the Trypan blue vital dye to study parasite viability. An aliquot of parasites were exposed to UV as a positive control of mortality. In contrast to the low percentage of survival displayed by UV irradiated parasites, survival of starved parasites was high and similar to the control (S1B Fig).

**Fig 1:**
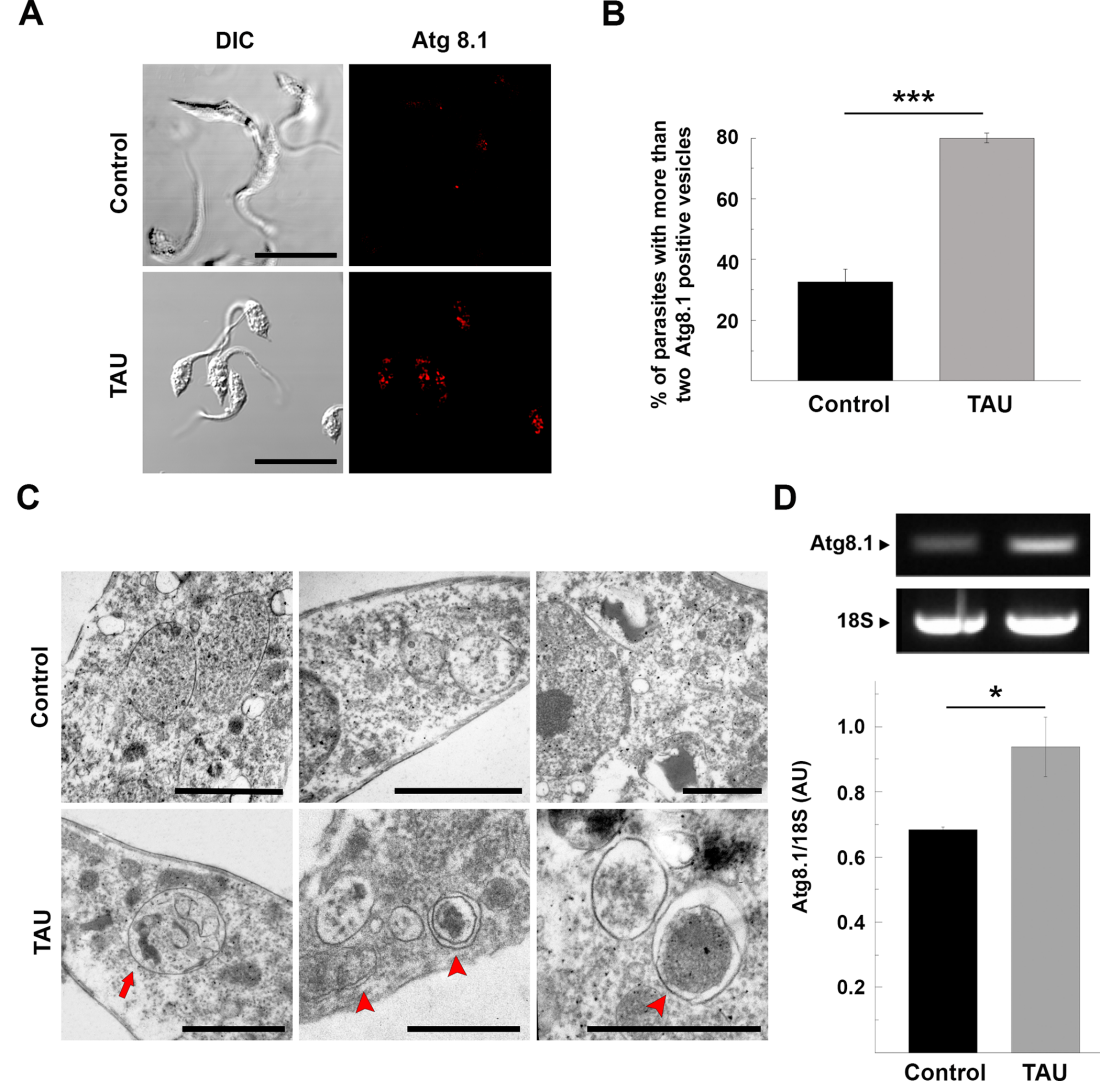
Autophagy is induced during the first period of metacyclogenesis. Y strain *T*. *cruzi* epimastigotes were incubated under control (BHT medium at 28°C) or starvation (TAU medium at 37°C) conditions for 2 h and then processed for either microscopy or molecular studies. **A:** Detection of the *Tc*Atg8.1 protein by IIF using a specific antibody. Confocal images depict autophagosomes labeled in red. Scale bar: 5 μm. **B:** Percentage of parasites with more than two Atg8.1 positive vesicles under each condition. Number of counted cells: 100. Data shown represent the mean +/- SE from 3 independent experiments. ***p < 0.001 (Tukey’s test). **C:** Autophagic structures visualized by TEM in the parasites subjected to starvation conditions (TAU) compared to control parasites (Control). The red arrow points to an amphisome and red arrowheads to the autophagosomes. Scale bar: 1 μm. **D:** The RT-PCR for *Tc*Atg8.1 was performed in cDNA obtained from parasites subjected to control or starvation conditions. The expression of *Tc*Atg8.1 was normalized to 18S rRNA expression and expressed as AU. Data shown represent the mean +/- SE from 3 independent experiments. **p<0.05 (Student’s *t* test).

Since maximal stress conditions were produced in the first 2 h, we tested autophagic response at this point, by studying the presence of autophagosomes. Parasites were fixed and processed for IIF. Autophagosomes could be readily detected in parasites subjected to nutritional and thermic stress, as compared to parasites maintained in full-nutrient media at 28°C (control conditions) (Fig 1A). Similar to the method described by Brady and coworkers, we quantified the number of Atg8.1 dots in parasites incubated under both conditions and empirically established a maximum threshold of two autophagosomes per parasite [26]. The percentage of parasites with more than two Atg8.1 positive vesicles was significantly higher in TAU (80 +/- 1.65%) than in control conditions (32.45 +/- 4.35%) (Fig 1B). Further TEM analyses of stressed parasites revealed the presence of double membrane vesicles and multivesicular compartments that resembled typical autophagic structures (Fig 1C). To confirm these observations, we studied the expression of *Tc*Atg8.1 by semi-quantitative RT-PCR using cDNA obtained from parasites maintained under control or TAU conditions. After data normalization to 18S rRNA, we observed that starved parasites presented a significant increase in the levels of *Tc*Atg8.1 cDNA, as compared to control parasites (Fig 1D).

**Fig 2:**
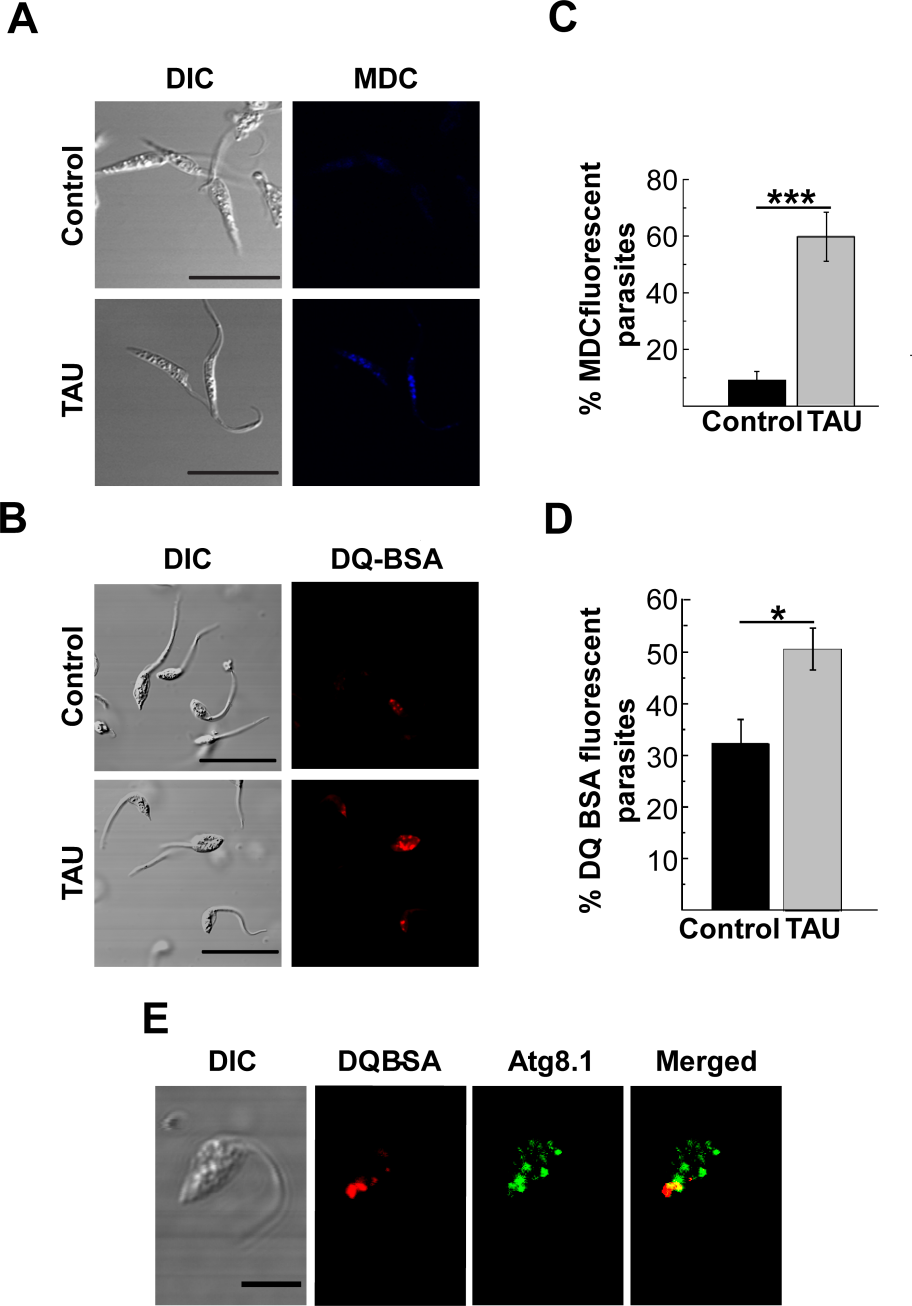
Characterization of acidic and degradative compartments during the first period of metacyclogenesis. Y strain *T*. *cruzi* epimastigotes were incubated under control (BHT medium at 28°C) or starvation (TAU medium at 37°C) conditions for 2 h and then processed to detect acidic or degradative compartments by microscopy. **A:** Parasites were stained with MDC and then analyzed by confocal microscopy *in vivo*. Acidic compartments were visualized in blue. Scale bar: 10 μm. **B:** Percentage of MDC fluorescent parasites incubated under each condition. Number of counted cells: 100. Data shown represent the mean +/- SE from 5 independent experiments. ***p < 0.001 (Tukey’s test). **C:** Parasites were stained with DQ-BSA and then analyzed by confocal microscopy *in vivo*. Degradative vesicles were visualized in red. Scale bar: 10 μm. **D:** Percentage of DQ-BSA positive parasites incubated under each condition. Number of counted cells: 100. Data shown represent the mean +/- SE from 3 independent experiments. *p < 0.05 (Tukey’s test). **E:** After the first period of metacyclogenesis, parasites were fixed and processed to detect Atg8.1 and DQ-BSA positive compartments as described in Methods. Confocal images depict the partial colocalization of autophagosomes (in green) and lysosomes (in red). Scale bar: 5 μm.

Apart from the autophagosome increase, the induction of autophagy in mammalian cells is characterized by an increase in the number of lysosomes/autolysosomes required for the lysis of trapped components [27]. Therefore, we used monodansylcadaverine (MDC) and the self-quenched albumin (DQ-BSA), which are markers of acidic and hydrolytic compartments respectively, to localize lysosomes in *T*. *cruzi*. As shown in Fig 2A and 2B epimastigotes maintained under nutrient-rich conditions displayed lower frequency of MDC or DQ-BSA labeling. In contrast, epimastigotes subjected to nutritional and thermic stress in TAU differentiation medium shown a significant increase in the number of lysosomes, as compared to controls (Fig 2C and 2D).

Similar differences were observed in the detection of MDC fluorescent intensity associated to parasites under each condition (S2 Fig).

In another set of experiments, we studied the colocalization of DQ-BSA and *Tc*Atg8.1 vesicles. We observed that starved parasites displayed a higher frequency of colocalization of DQ-BSA with *Tc*Atg8.1, as compared to controls (Mander´s overlap coefficient 0.87 +/- 0.03 vs. 0.42 +/- 0.05), indicating fusion of autophagosomes with lysosomes to form autolysosomes (Fig 2E).

Taken together, these results evidence that nutritional and thermic stress conditions induce the autophagic pathway during the *T*. *cruzi* metacyclogenesis.

### Role of main autophagy modulators on *T*. *cruzi* autophagy

**Fig 3:**
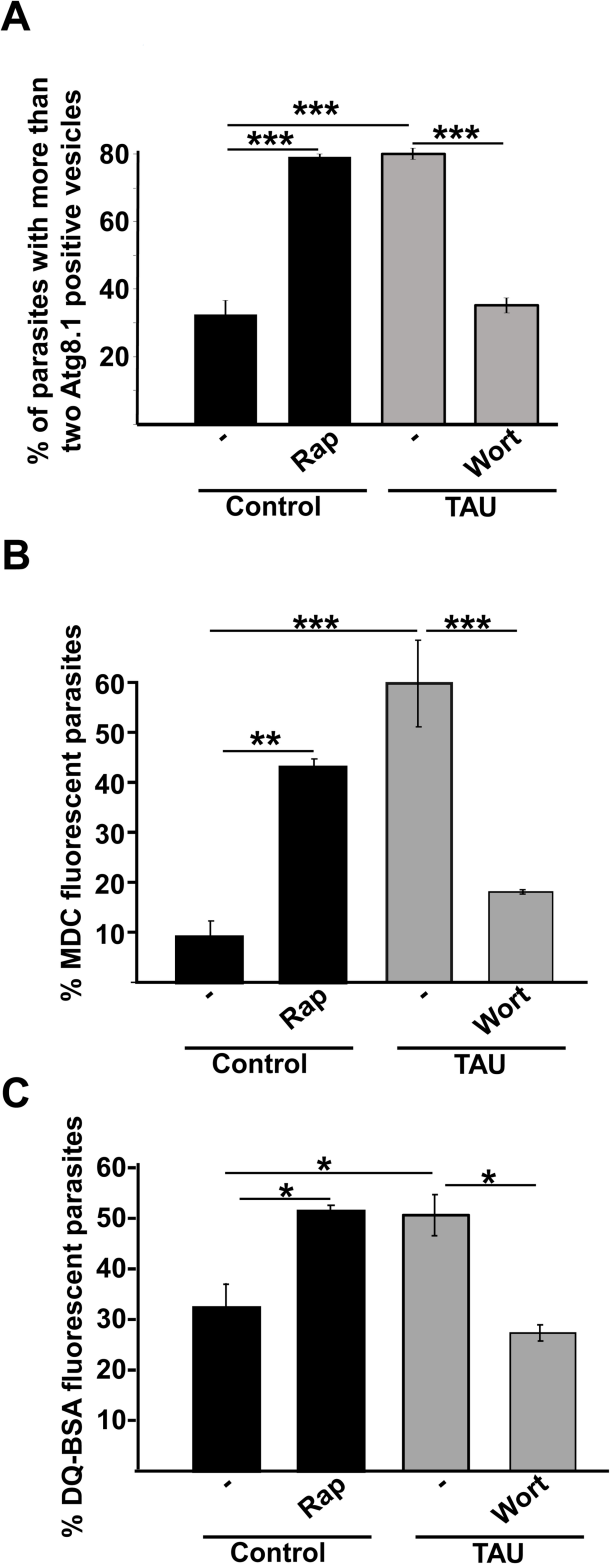
Effect of autophagy modulators on *T*. *cruzi* autophagy. Y strain *T*. *cruzi* epimastigotes were incubated in control medium in the absence or the presence of 50 ng/μl rapamycin (Rap) at 28°C or in TAU medium in the absence or the presence of 100 nM wortmannin (Wort) at 37°C for 2 h. After these treatments, parasites were processed to detect autophagosomes through the presence of *Tc*Atg8.1 **(A)**, MDC staining showing the presence of acidic autophagosomes **(B),** DQ-BSA staining depicting degradative compartments by confocal microscopy **(C)** (see details in Methods). The percentage of parasites labeled with these markers under each condition was quantified. Number of counted cells: 100. Data shown represent the mean +/- SE from 3 independent experiments. * p < 0.05, ** p < 0.01, ***p < 0.001 (Tukey’s test).

Next, we studied the possible participation of mTOR and Vps34 kinases on *T*. *cruzi* autophagy. As shown in Fig 3A, the treatment of parasites with 50 ng/μl Rap under control conditions (Diamond media at 28°C) for 2 h, induced a significant increase in the percentage of parasites with more than two Atg8.1 positive vesicles, as compared to non-treated parasites. This increment in the autophagic response was similar to that obtained in the first period of differentiation in parasites incubated in TAU medium. Conversely, treatment with 100 nM Wort of parasites exposed to differentiation conditions impaired the autophagic response (Fig 3A). Similar differences were observed in the content of acidic and hydrolytic vesicles detected with MDC and DQ-BSA, respectively (Fig 3B and 3C).

**Fig 4:**
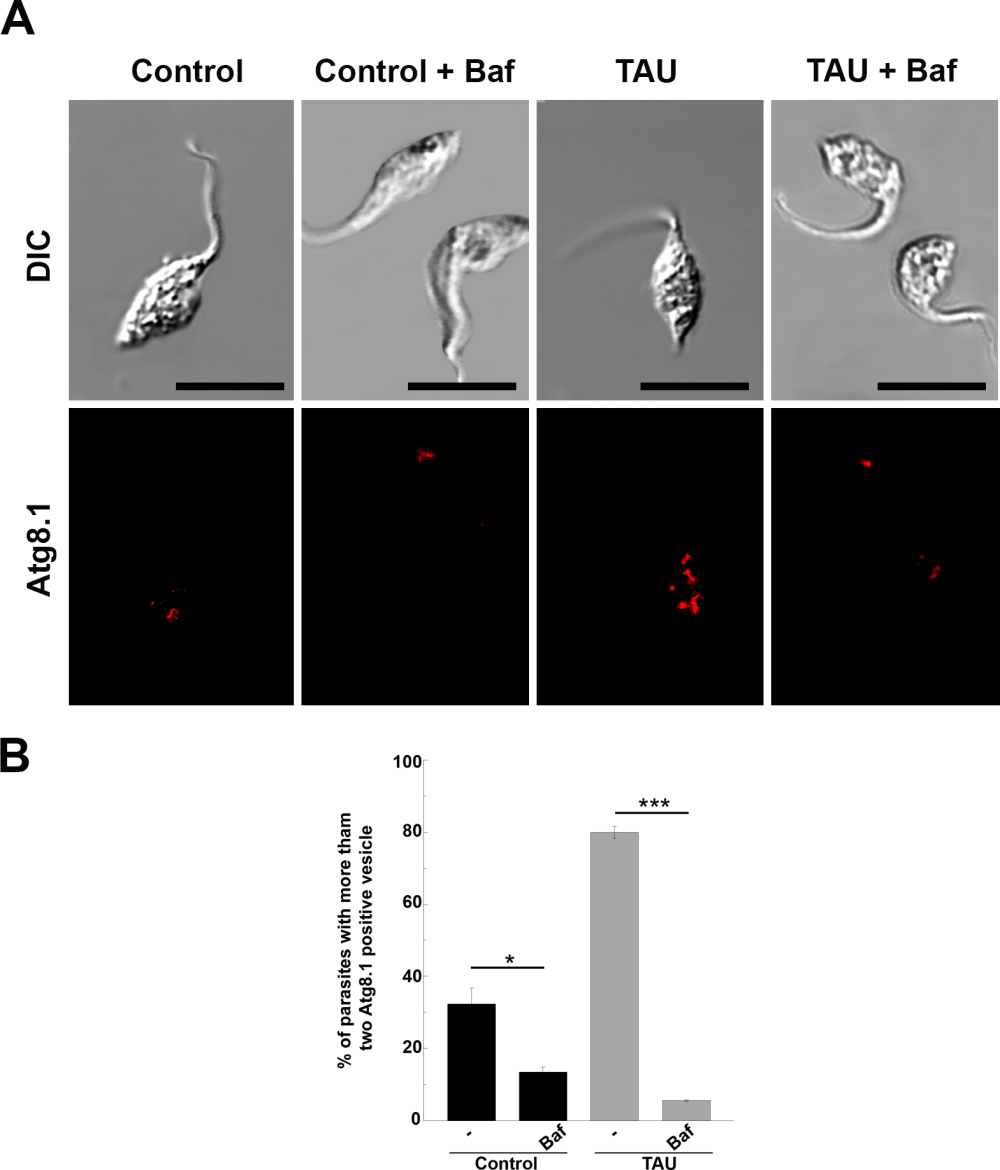
Effect of bafilomycin on parasites after the first period of metacyclogenesis. Y strain *T*. *cruzi* epimastigotes were incubated under either control (Control) or starvation (TAU) conditions in the absence or the presence of 100 nM of bafilomycin (Baf) for 2 h and then processed to observe autophagosomes for IIF using the *Tc*Atg8.1 antibody. **A:** Confocal images depict autophagosomes labeled in red. Scale bar: 10 μm. **B:** Percentage of parasites with more than two Atg8.1 positive vesicles under each condition. Number of counted cells: 100. Data shown represent the mean +/- SE from 3 independent experiments ** p < 0.05, ***p < 0.001 (Tukey’s test).

Bafilomycin (Baf), which is another important autophagy inhibitor, is mainly used to study the autophagic flux [28]. The treatment of mammalian cells with Baf blocks the normal autolysosomal degradation, leading to accumulation of autophagic compartments at different maturation stages. Unexpectedly, a significant reduction in the autophagosome number was observed when parasites were incubated in TAU in the presence of Baf, as compared to TAU medium alone (Fig 4A and 4B).

The number of autophagosomes was lower in parasites subjected to Baf in control medium than in non-treated parasites, indicating that Baf inhibits both basal and induced autophagy. Unlike the effect observed in mammalian cells, Baf abrogated autophagosome formation in *T*. *cruzi*, resulting in a complete inhibition of the autophagic response.

### Polyamine metabolism and autophagy in *T*. *cruzi*

**Fig 5:**
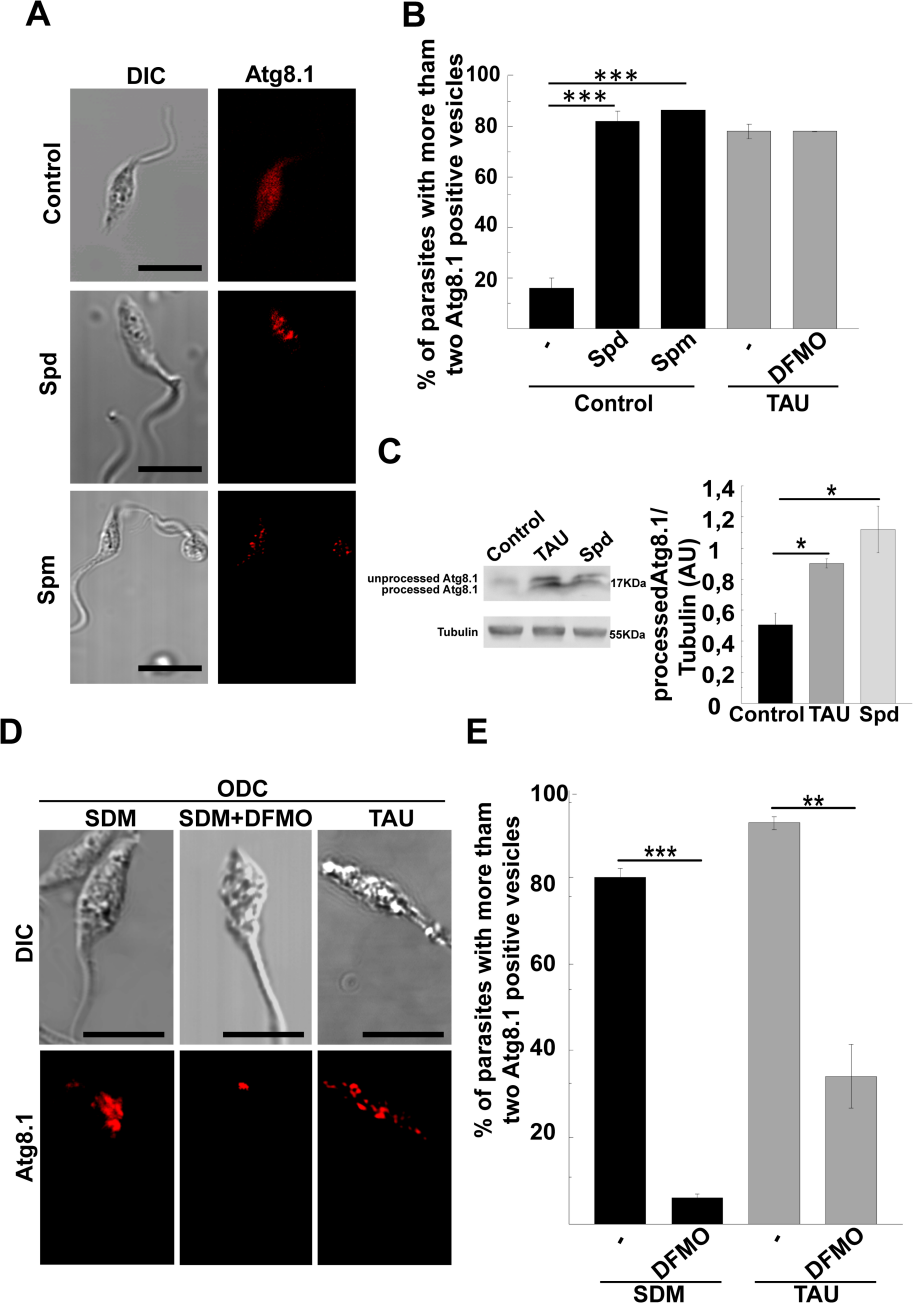
Effect of polyamines on *T*. *cruzi* autophagy. Y strain *T*. *cruzi* epimastigotes were incubated under control conditions in the absence or the presence of 100 μM of spermidine (Spd) or 100 μM of spermine (Spm); or under starvation conditions in either the absence or the presence of 1 mM of DFMO for 2 h and then processed for autophagosome detection through *Tc*Atg8.1 by IIF. **A:** Confocal images depict autophagosomes labeled in red at the indicated conditions. Scale bar: 10 μm. **B:** Percentage of parasites with more than two Atg8.1 positive vesicles. Number of counted cells: 100. Data shown represent the mean +/- SE from 3 independent experiments. ***p < 0.001 (Tukey’s test)**. C:** Expression of *Tc*Atg8.1 by western blot. Protein extracts were obtained from parasites maintained under the indicated conditions and used to detect *Tc*Atg8.1 by western blot (see details in Methods). The expression of *Tc*Atg8.1 was normalized to tubulin and expressed as AU. Data shown represent the mean +/- SE from 3 independent experiments ** p < 0.05 (Tukey’s test). **D:** Mutant Y-GFP-ODC epimastigotes were incubated in SDM or TAU media in either the absence or the presence of 1 mM of DFMO for 2 h and processed as above. Confocal images depicting the level of autophagosomes labeled with the *Tc*Atg8.1 protein (red) at the indicated conditions. **E:** Percentage of parasites with more than two Atg8.1 vesicles. Number of counted cells: 100. Data shown represent the mean +/- SE from 3 independent experiments. * p < 0.01, ***p < 0.001 (Tukey’s test).

Polyamines (PA) are low molecular mass polycations that bind to acidic macromolecules such as DNA, RNA and proteins to regulate proliferation and differentiation. Ornithine decarboxylase 1 (ODC1), which is one of the rate-limiting enzymes in the polyamine biosynthetic pathway, catalyzes the conversion of L-ornithine to putrescine (Put). The sequential addition of two aminopropyl groups to Put by spermidine synthase and spermine synthase generates spermidine (Spd) and spermine (Spm), respectively. Since the role of Spd on *T*. *cruzi* autophagy has not been studied before; we analyzed the effect of Spd and also Spm in our system. Our results showed that the presence of either Spd or Spm significantly increased the detection of Atg8.1 (Fig 5A). Quantitative data showed a significant increase of parasites with autophagic vesicles after PA treatment, as compared to control parasites (≈ 80% *vs*. ≈ 20%). Interestingly, the degree of autophagic response of *T*. *cruzi* in the presence of PA was similar to that obtained under TAU condition. Furthermore, addition of 1mM DFMO, which is a non-reversible inhibitor of ODC [29], to TAU, did not modify the number of autophagosomes, as compared to TAU condition alone (Fig 5B). This result was not surprising due to the lack of ODC1 in *T*. *cruzi* [30].

As demonstrated previously, *T*. *cruzi* present all components of the Atg8 conjugation system [13]. Therefore, induction of autophagy in the parasite involves the processing and lipidation of *Tc*Atg8.1 to insert it in the membrane of the autophagosome. Next we performed a western blot assay to detect the two different forms (soluble and membrane-bound) of *Tc*Atg8.1 from protein extracts obtained of parasites subjected to control or autophagic-induced conditions. As shown in Fig 5C, a double band corresponding to unprocessed *Tc*Atg8.1 (soluble) and processed *Tc*Atg8.1 (membrane-bound) were detected in TAU and Spd conditions while parasites maintained in control medium contain only the soluble form. Level of processed *Tc*Atg8.1 normalized to tubulin was significantly increased under TAU and Spd treatments, as compared to control, thus confirming the processing of this protein when autophagy is triggered.

As mentioned above, *T*. *cruzi* is unable to synthesize endogenous PA due to the lack of ODC [30], thus relying on PA uptake from the extracellular medium by the polyamine permease *Tc*PAT12 [23,30]. In our laboratory, we have previously generated a *T*. *cruzi* mutant strain coexpressing heterologous ODC and GFP (Y-GFP-ODC) [22] that reverted the natural polyamine auxotrophy. In this work, we have used this strain to confirm the effect of PA on parasite autophagy. To ensure ODC activity, Y-GFP-ODC parasites were maintained in the semisynthetic medium SDM79 (containing only traces of polyamines). In this medium, ≈ 80% of parasites displayed more than two Atg8.1 positive dots (Fig 5D and 5E). The number of autophagosomes increased when mutant parasites were subjected to the first period of differentiation in TAU medium at 37°C. In contrast, the treatment of parasites with 1 mM DFMO in SDM or TAU media significantly reduced the autophagic response.

Taken together, these results demonstrated that PA are important inducers of autophagy in *T*. *cruzi* and that the higher basal autophagy of Y-GFP-ODC parasites was due to the activity of heterologous ODC and the increased availability of PA. Similar conclusions were obtained with a mutant strain that overexpresses the *Tc*PAT12 transporter and displays a higher uptake of PA [31]. As expected, in SDM79 medium, only 30% of Y-GFP-PAT12 parasites displayed autophagic activity. This percentage increased significantly after addition of 100 μM Spd or 100 μM Spm to the medium or when parasites were subjected to differentiation conditions in TAU medium. Unexpectedly, the presence of DFMO reduced the basal autophagy levels in this strain, probably by an indirect effect of the drug (S3 Fig).

### Induction of autophagy promotes *T*. *cruzi* metacyclogenesis

**Fig 6:**
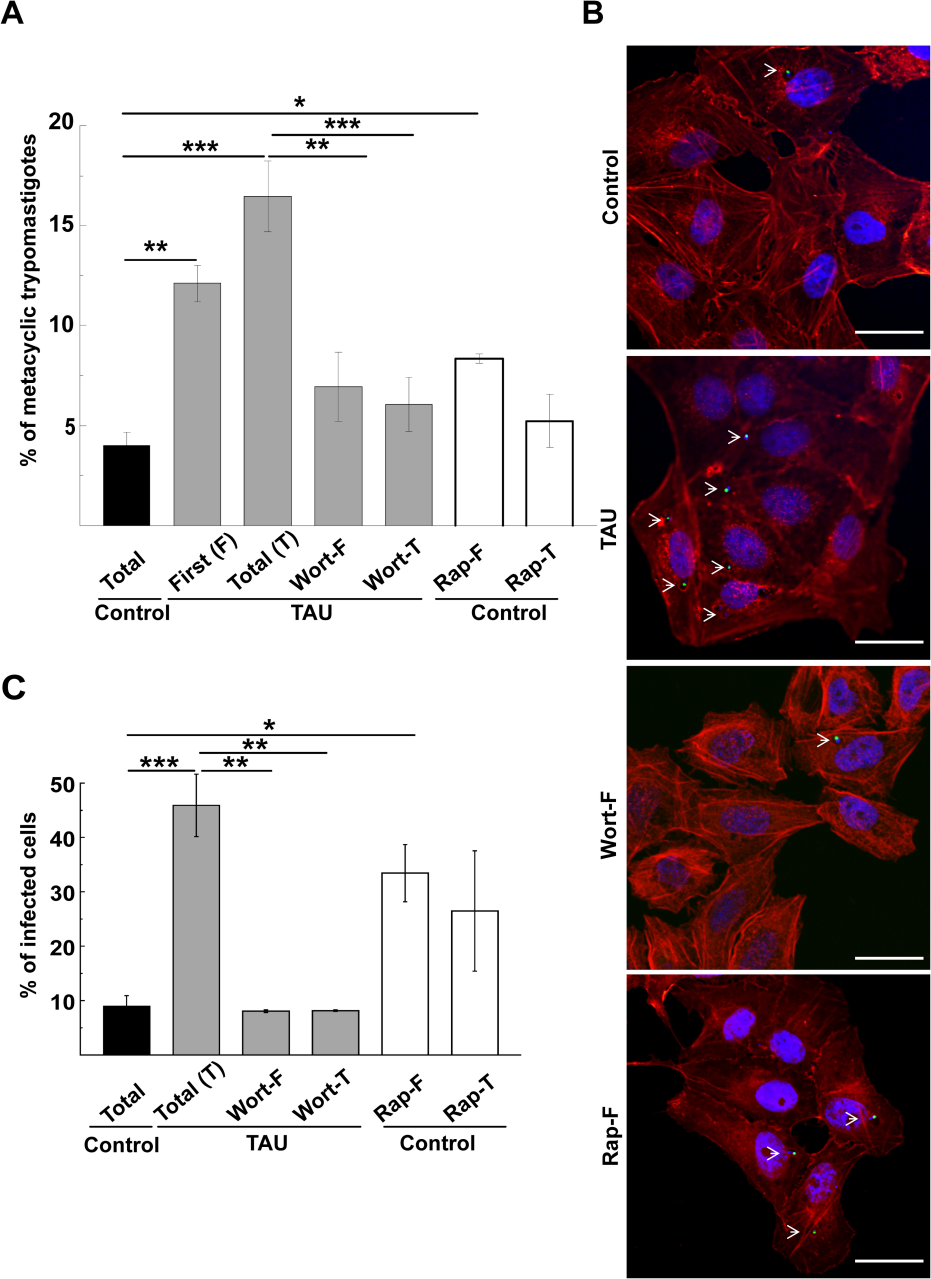
Autophagy induction promotes metacyclogenesis in *T*. *cruzi*. Y-GFP strain *T*. *cruzi* epimastigotes were subjected to the first (F) or the total (T) period of metacyclogenesis in control medium in either the absence or the presence of 50 ng/μl of rapamycin (Rap) or in TAU medium in either the absence or the presence of 100 nM of wortmannin at 37°C for 2 h. **A:** Trypomastigotes generated under each condition. Number of counted cells: 15 x 10^6^ cells. Data shown represent the mean +/- SE from 3 independent experiments. ** p < 0.01, ***p < 0.001 (Tukey’s test). **B:** Equivalent samples were placed over Vero cell monolayers for 24 h to allow infection and then fixed and processed as described in Methods. Confocal images show cells **i**nfected with MT generated under different conditions (actin was visualized in red by phalloidin-rhodamine staining, whereas *T*. *cruzi* amastigotes were observed in green). Scale bar: 10 μm **C:** Percentage of infected cells at the indicated conditions. Number of counted cells: 100. Data shown represent the mean +/- SE from 3 independent experiments. * p < 0.05, ** p < 0.01, ***p < 0.001 (Tukey’s test).

Next, we studied the effect of the modulation of autophagy on the global process of metacyclogenesis. Y-GFP strain *T*. *cruzi* epimastigotes were subjected to the complete differentiation process in either the presence of Rap or Wort in either the first (F) or the total (T) period. After this time, samples were treated with human fresh serum to induce the complement-dependent lysis of epimastigotes. Generated MT were then directly observed and counted in a Neubauer chamber (S1 Fig, see details in Methods). Our results showed that the percentage of MT differentiated up to 48 h was higher in both TAU medium at the first (TAU-F) and the total (TAU-T) period of metacyclogenesis than in controls (12.1 +/- 0.9% and 16.4 +/- 1.7% vs. 3.9 +/- 0,6%, Fig 6A). Furthermore the addition of Wort to TAU during the first (Wort-F) or the total (Wort-T) period significantly reduced the number of MT in the parasite mixture, as compared to TAU-T condition. Conversely, Rap was able to increase the generation of MT when added to control medium at the first period of differentiation (8.3 +/- 0.2% vs. 3.9 +/- 0.6%). In contrast, Rap had not effect when added to the total period due to a toxic action of this compound on parasites at longer times. In another set of experiments, samples were allowed to infect Vero cells monolayers during 24 h and then fixed and processed for confocal microscopy. The degree of cell infection was considered an indirect measure of the number of MT present in the samples at different conditions, as previously described [22]. As depicted in Fig 6B, parasite nuclei were visualized in green due to the stable expression of *Tc*H2b histone fused to GFP (24), whereas host cells were in red due to the labeling with phalloidin-rhodamine that binds to actin cytoskeleton. The blue color depicts cell and parasite nuclei and parasite kinetoplast stained with Hoechst. Results showed that around 45% of cells were infected by TAU differentiated parasites, whereas 10% of the cells were infected by control parasites. The presence of Wort at either the first (Wort-F) or the whole (Wort-T) period of differentiation in TAU significantly reduced the infection rate. In concordance to the above result, the addition of Rap at the first period of time (Rap-F) induced a significant increase of infectivity (Fig 6C).

Taken together, these results demonstrated that autophagy has a key role during *T*. *cruzi* differentiation. Therefore, modulation of autophagy can be used to interrupt the metacyclogenesis rate in *T*. *cruzi* and to block the normal progression of the parasite biological cycle.

## Discussion

Metacyclogenesis is an essential process for the transmission of *T*. *cruzi* from the insect vector to mammalian hosts, which involves several metabolic and morphological changes in the parasite. Although the relationship between the rate of metacyclogenesis and a low nutritional state of the vector was described many years ago [32], the specific cellular response to this stimulus was solely recognized upon identification of *T*. *cruzi* ATG genes [13]. In this work, we applied a previously published method of *in vitro* differentiation [20,25] to make a systematic analysis of autophagy and its possible modulators during *T*. *cruzi* metacyclogenesis.

Our data show that after the first two hours of metacyclogenesis induction (S1 Fig), there is an increased autophagic activity in epimastigotes, evidenced by a higher number of autophagosomes and lysosomes observed by both fluorescence and electron microscopy. A higher autophagic response of parasites is achieved during this first period of differentiation, when epimastigotes were exposed to a severe nutritional and thermic stress. Metacyclogenesis naturally occurs in the gut of the insect vector after a period of rapid multiplication of epimastigotes. Several factors are required to activate this process. Attachment of the parasite to the luminal surface of the insect’s rectum, hemolymph components, cAMP action and even the redox status of the parasite may promote metacyclogenesis [33–37]. Similarly to our *in vitro* method, a starvation environment caused by the higher number of replicating epimastigotes is another important inductor of metacyclogenesis. Considering that the main cellular response to starvation is autophagy, it is was not a surprise that this process was activated during parasite differentiation, as previously observed by other authors [18].

As mentioned above, metacyclogenesis is characterized by a renewal of proteins and subcellular structures required for parasite infection of a new host, while eliminating others that are no longer needed. It is expected that the pathways of degradation play a very important role and, among them, the autophagic pathway. During differentiation of the protozoan pathogen *Trypanosoma brucei* from the bloodstream form to the procyclic trypomastigote, the glycosomes, which are organelles that contain the enzymes of the glycolytic pathway, are significantly reduced while new organelles containing different enzymes are synthesized [38]. Further experiments carried out by the same research group have demonstrated that this efficient glycosome turnover involves autophagic degradation and confer to procyclic trypomastigotes the capacity to survive under the low glucose environment of the mosquito’s salivary glands [39]. Similarly to *T*. *brucei*, *T*. *cruzi* exhibits morphological and biochemical changes among the different stages. In this sense, previously works have evidenced a massive proteolysis during *T*. *cruzi* differentiation [40,41]. In agreement with these data, we observed that during the induction of metacyclogenesis, there occurs a significant expansion of the lysosomal compartment, as demonstrated by the increased number of acidic and hydrolytic vesicles. Moreover, the high colocalization levels of DQ-BSA and Atg8.1 indicates that the autophagic activity contribute to this process. Many virulence factors expressed in the infective forms may be modified by this increased hydrolytic activity. The trans-sialidase family member gp82 that is expressed earlier during differentiation is located in cruzipain-positive organelles at the posterior region before it is delivered towards the cell surface [42]. Autophagic degradation may also contribute to the cytostome-cytopharynx disappearance and the loss of the endocytic ability observed at the end of metacyclogenesis [43].

We next studied the effect of drugs widely used to modulate mammalian autophagy. Our data showed that rapamycin and wortmannin stimulated and inhibited parasite autophagy, respectively, indicating that the molecular targets of these drugs also exist in *T*. *cruzi*. Rapamycin is a reversible inhibitor of the kinase mTOR, a central regulator of cell growth [44]. Besides a study of mammalian TOR inhibitors as repurposed drugs against kinetoplastid parasites [45], this is the first report that describes the cellular effects of rapamycin in *T*. *cruzi*. In *T*. *brucei*, TOR kinases are an extended family of proteins comprising the *Tb*TOR1 and *Tb*TOR2 that form the complexes TORC1 and TORC2 similar to mammals, and two additional TOR kinases, *Tb*TOR3 and *Tb*TOR4. The latter forms a third complex that negatively regulates parasite differentiation [46], whereas the *Tb*TORC2 complex participates in cell growth and is sensible to rapamycin [47]. Ortholog sequences with homology to *Tb*TOR and other genes that encode proteins with putative domains of TOR kinases were detected in *T*. *cruzi*. Three of them also contain the rapamycin recognizing domain [48]. Although the exact number and function of putative *Tc*TOR genes is still unknown, our experimental data demonstrate a clear effect of Rap as an inducer of parasite autophagy and, as a consequence, of metacyclogenesis.

Classical autophagy inhibitors like wortmannin have an effect on *T*. *cruzi* autophagy and metacyclogenesis. The presence and activities of inositol kinases in *T*. *cruzi* epimastigotes have been previously characterized [49]. The *Tc*Vps34 kinase plays an important role in osmoregulation, acidification and vesicular trafficking [50] and, as demonstrated in this work, also as an autophagy inhibitor. This enzyme is regulated by the *Tc*Vps15 catalytic activity and both *Tc*Vps15-*Tc*Vps34 form a complex that partially colocalizes to autophagosomes [51]. On the other hand, and contrarily to the autophagosome accumulation observed in mammalian cells after Baf treatment, this compound abrogated autophagosome formation in *T*. *cruzi*, resulting in a complete inhibition of the autophagic response after TAU treatment. This is probably a similar phenomenon to that observed in *T*. *brucei*. In this parasite, the acidocalcisome, which is a lysosome-related organelle characterized by acidic pH and a high content of Ca^2+^ and polyphosphates, has been found to regulate autophagy. Li et al. have demonstrated that the induction of autophagy in *T*. *brucei*, is accompanied by an acidification of acidocalcisomes and that drugs that impair this process, such as bafilomycin, completely inhibit the formation of autophagosomes [52]. Even though the mechanism has not been fully elucidated yet, the acidification of acidocalcisomes upon starvation of parasites, activates the synthesis of PI3P in the membrane of these organelles, a process required for autophagosome biogenesis [53].

In this work, we also verified that spermidine and spermine, that may act under some circumstances as a Spd analogue [31,54,55], exerting a potent induction of *T*. *cruzi* autophagy under control conditions. The effect of Spd on autophagy has previously been demonstrated in yeast, flies, worms, and human immune cells [9]. In those cases, the activation of autophagy prolonged the life span of those organisms by counteracting the aging processes [10]. In contrast, on *T*. *cruzi*, and as previously shown [22], PA activated differentiation processes like metacyclogenesis. In this work, we demonstrate that this action is mediated by the induction of parasite autophagy. Since the spermidine action is related to an increased expression of ATG genes [9], and given the *T*. *cruzi* auxotrophy for polyamines, the acquisition of PA by the parasite should be produced before autophagy induction. For this reason, mutant parasites that expressed the heterologous ODC gene displayed high basal autophagic activity that was suppressed by DFMO, the non-reversible inhibitor of ODC. Moreover, when these mutants were incubated in TAU, autophagy was even higher, indicating an additive effect of both starvation and PA on the induction of autophagy. As expected, in contrast to ODC, the PAT12 mutant did not exhibit high basal autophagy until Spd or Spm were present in the medium (S3 Fig). Surprisingly DFMO was able to inhibit basal autophagy in this mutant. An explanation of this unexpected result is the possible existence of an interference with the transport of PA (or amino acids) in the presence of the drug. Further studies are needed to clarify this point.

Similarly to metacyclogenesis, other processes of *T*. *cruzi* differentiation may require autophagic activity. In a previous work we have found high levels of Atg8 in amastigotes located in the host cell cytoplasm, indicating the existence of increased autophagic activity in parasites at this stage [56]. Other works have also demonstrated the key role of protein degradation during amastigogenesis [16,57]. A detailed study of such mechanisms together with their possible inhibitors are crucial to find new drugs that interrupt the life cycle of *T*. *cruzi*. On the other hand, many trypanocidal drugs trigger autophagy in the parasite [58,59]. In this context, a deep knowledge of the mechanisms that regulate autophagy in this pathogen will contribute to a better understanding of the mechanisms of action of these drugs and improve the strategies for the treatment of Chagas’ disease.

## Supporting information

S1 Fig **A:** Scheme of the method of *T*. *cruzi in vitro* differentiation from epimastigotes to metacyclic trypomastigotes (metacyclogenesis). **B:** Parasite viability was controlled after the first period of metacyclogenesis by the Trypan blue dye exclusion method and expressed as percentage of parasite survival in control (Control), starvation (TAU) and after UV irradiation (UV).
(TIF)

S2 Fig MDC fluorescent intensity associated with Y-GFP strain *T*. *cruzi* epimastigotes incubated under control (Diamond medium at 28°C) or starvation (TAU medium at 37°C) conditions for 2 h. After MDC labeling, fluorescent intensity was measured by spectrofluorometry and expressed as arbitrary units (AU). Data shown represent the mean +/- SE from 3 independent experiments. * p < 0.05 (Student’s *t*-test).
(TIF)

S3 Fig Y-PAT 12 mutant *T*. *cruzi* epimastigotes were incubated in SDM in either the absence (SDM) or the presence of 1 mM of DFMO (SDM + DFMO), 100 μM of spermidine (SDM + Spd) or 100 μM of spermine (SDM + Spm) at 28°C for 2 h and then processed to observe autophagosomes by detection of *TcAtg8*.1 protein by IIF. Another sample of epimastigotes was incubated with TAU medium as a control of autophagic induction and processed as before. **A:** Confocal images depict autophagosomes labeled in red at the indicated conditions. Scale bar: 10 μm. **B:** Percentage of parasites with more than two Atg8.1 positive vesicles. Number of counted cells: 100. Data shown represent the mean +/- SE from 3 independent experiments. ** p < 0.01, ***p < 0.001 (Tukey’s test).
(TIF)
