# Supplementary figures and images for: The regulation of autophagy differentially affects Trypanosoma cruzi metacyclogenesis
Source: PLoS Negl Trop Dis. 2017 Nov 1;11(11):e0006049. doi: 10.1371/journal.pntd.0006049 (PMC5683653; doi:10.1371/journal.pntd.0006049)

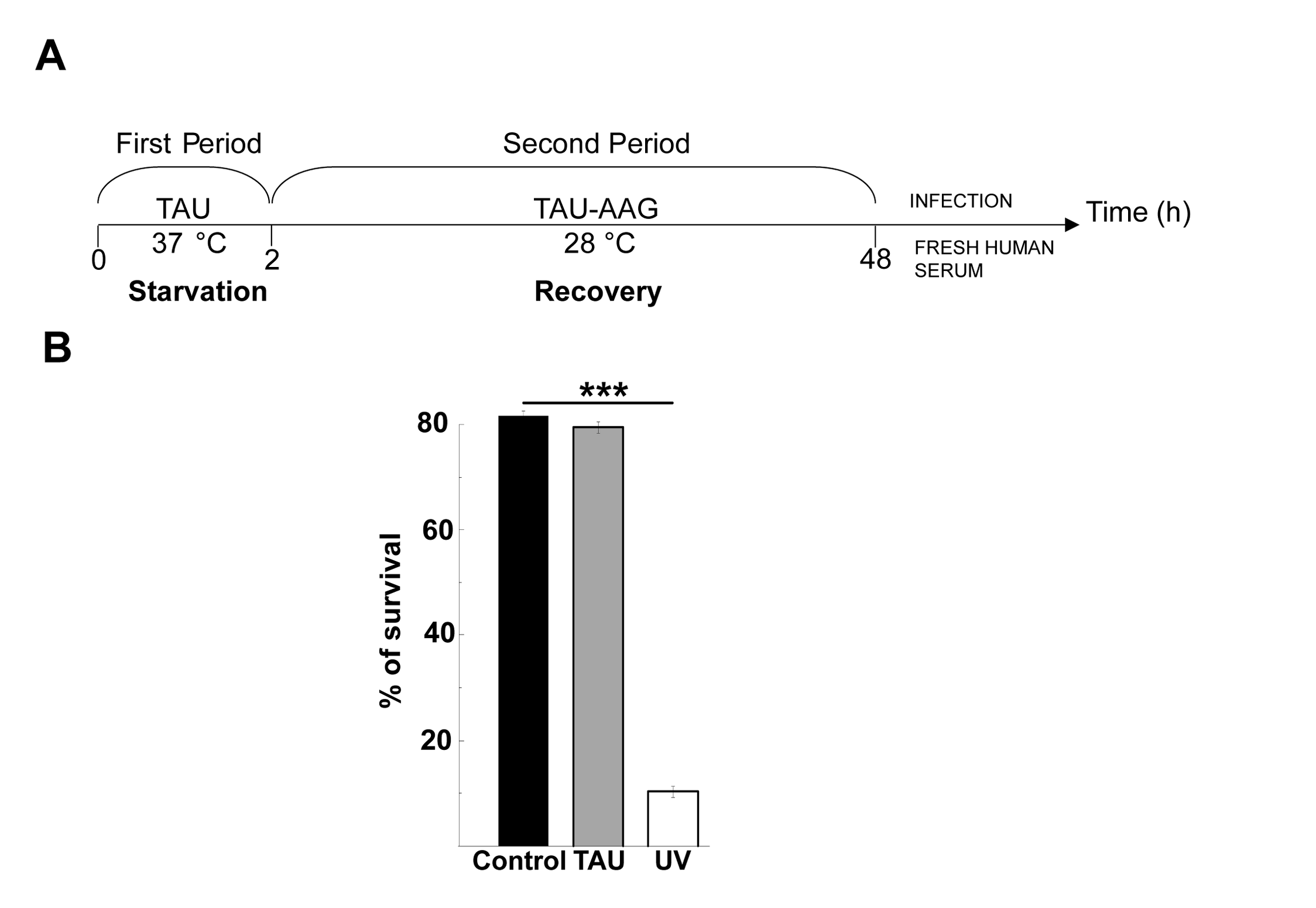

Supplement: S1 Fig — A: Scheme of the method of T. cruzi in vitro differentiation from epimastigotes to metacyclic trypomastigotes (metacyclogenesis). B: Parasite viability was controlled after the first period of metacyclogenesis by the Trypan blue dye exclusion method and expressed as percentage of parasite survival in control (Control), starvation (TAU) and after UV irradiation (UV). (TIF) [file pntd.0006049.s001.tif]

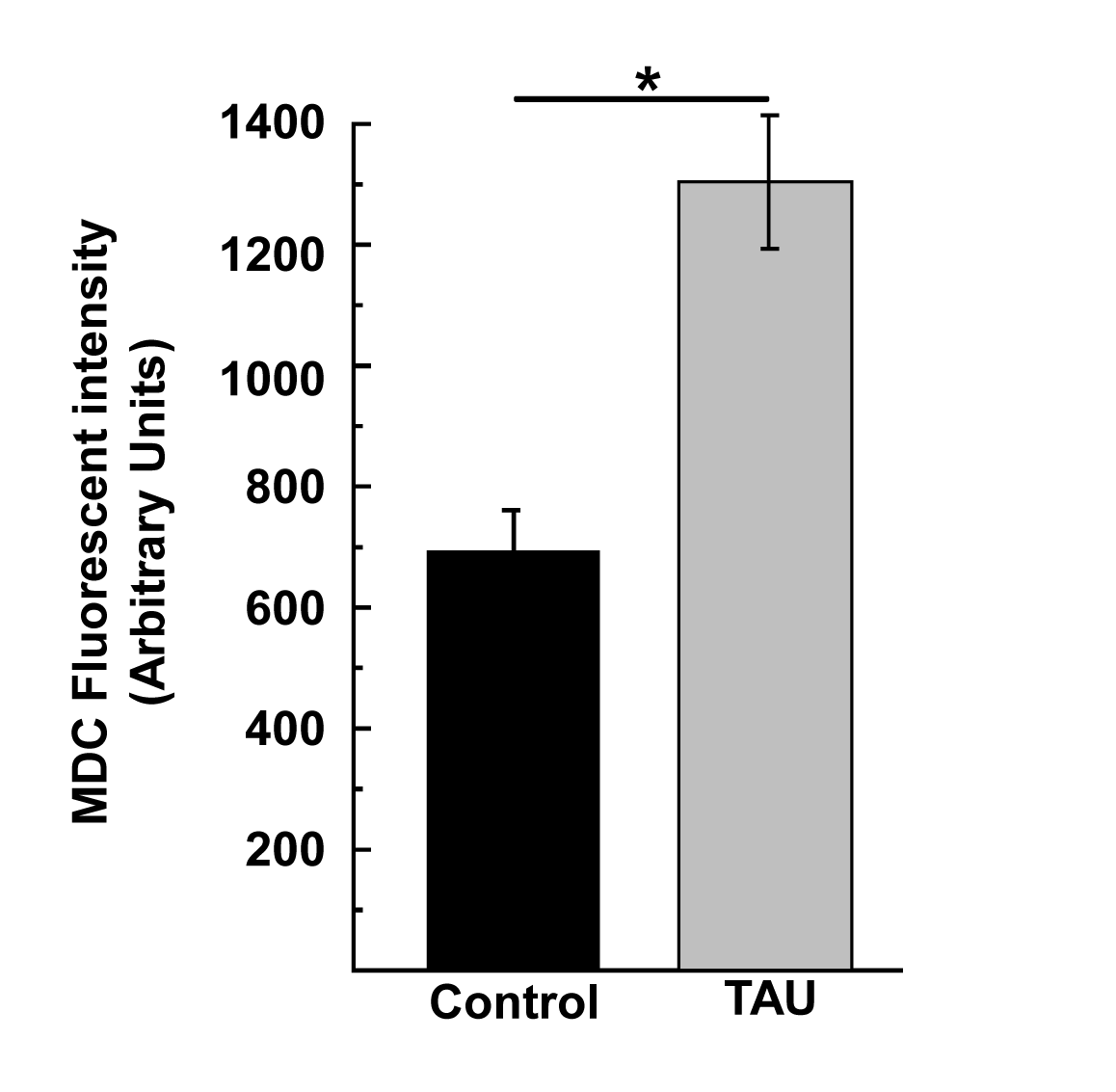

Supplement: S2 Fig — After MDC labeling, fluorescent intensity was measured by spectrofluorometry and expressed as arbitrary units (AU). Data shown represent the mean +/- SE from 3 independent experiments. * p < 0.05 (Student’s t-test). (TIF) [file pntd.0006049.s002.tif]

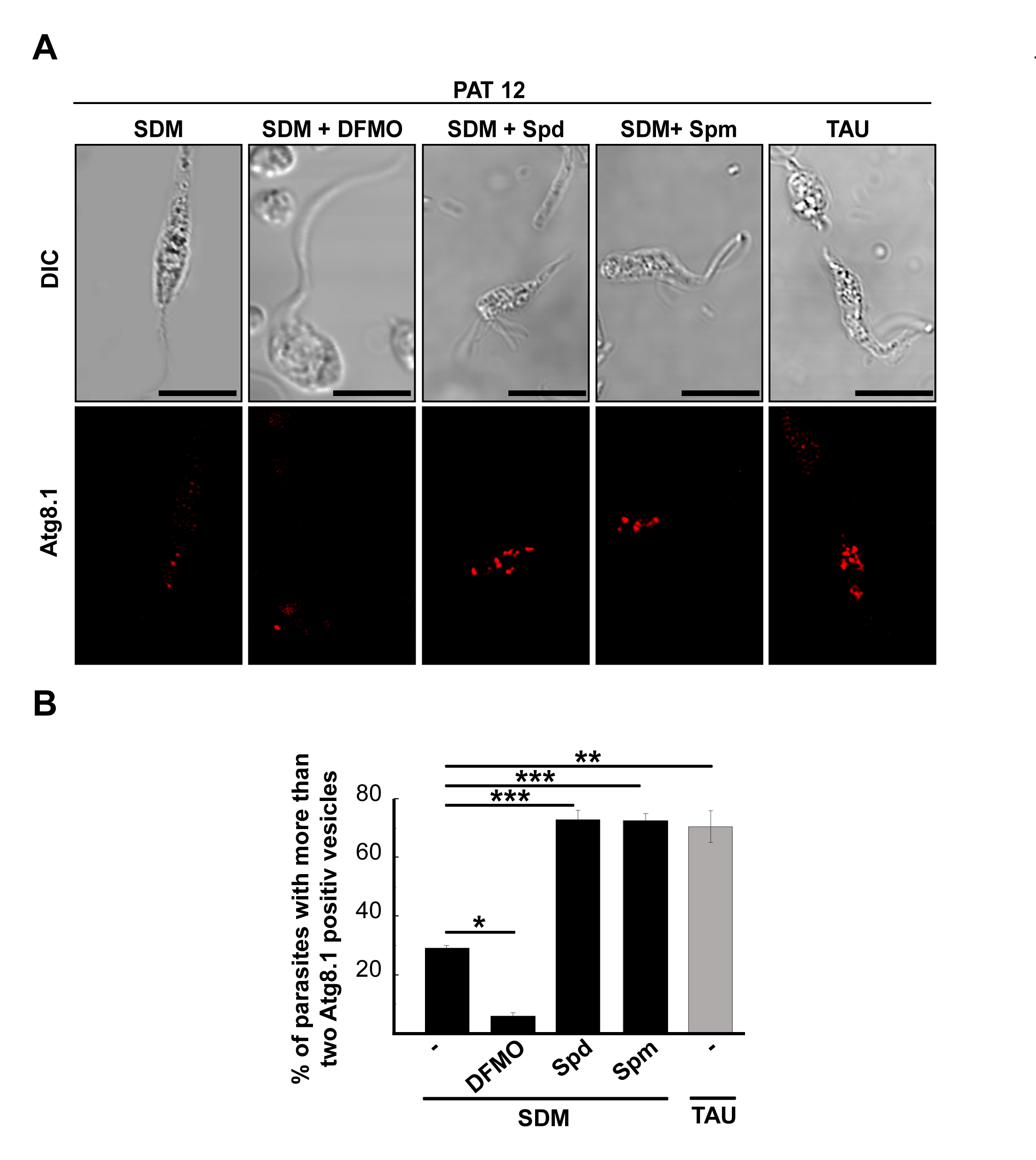

Supplement: S3 Fig — Another sample of epimastigotes was incubated with TAU medium as a control of autophagic induction and processed as before. A: Confocal images depict autophagosomes labeled in red at the indicated conditions. Scale bar: 10 μm. B: Percentage of parasites with more than two Atg8.1 positive vesicles. Number of counted cells: 100. Data shown represent the mean +/- SE from 3 independent experiments. ** p < 0.01, ***p < 0.001 (Tukey’s test). (TIF) [file pntd.0006049.s003.tif]
